# Comparison of postoperative corneal optical density changes and influencing factors between SMILE and FS-LASIK for high myopia: A retrospective observational study

**DOI:** 10.1097/MD.0000000000045117

**Published:** 2025-10-10

**Authors:** Jing Lou, Yue Xu, Li Wang, Yun Wang, Yao Xu, Xiaofeng Zhang

**Affiliations:** a Department of Ophthalmology, the Fourth Affiliated Hospital of Soochow University (Suzhou Dushu Lake Hospital), Suzhou, China.

**Keywords:** corneal optical density, femtosecond laser–assisted keratomileusis, high myopia, influencing factors, small incision lenticule extraction

## Abstract

To compare postoperative changes in corneal optical density (COD) and their influencing factors between small incision lenticule extraction (SMILE) and femtosecond laser-assisted in situ keratomileusis (FS-LASIK) in patients with high myopia. A retrospective analysis was conducted on patients with high myopia who underwent SMILE (n = 132 eyes) or FS-LASIK (n = 147 eyes). COD measurements were obtained using a Scheimpflug camera at baseline and at 1-day, 1-week, 1-month, 3-months, and 6-months postoperatively. COD was evaluated in the 0 to 2, 2 to 6, 6 to 10, and 10 to 12 mm regions. Changes in COD across different time points and regions were compared between the 2 groups. Both groups demonstrated a decreasing trend in average COD at 6-months postoperatively. Changes in COD were influenced by the surgical type, corneal region, and time point (all *P* < .05). In the 0 to 2 and 2 to 6 mm regions, COD at 1-week and 1-month postoperatively was higher in the SMILE group compared to the FS-LASIK group (all *P* < .05). In the 0 to 2 mm region, baseline COD and cap/flap thickness were identified as factors influencing COD changes at 1-week postoperatively, while baseline COD was the sole factor at 1-month postoperatively (all *P* < .05). In the 2 to 6 mm region, baseline COD was a factor influencing COD changes at 1-week postoperatively in the SMILE group, whereas flap thickness was the main factor in the FS-LASIK group. At 1-month postoperatively, baseline COD was the only influencing factor in both groups (all *P* < .05). Postoperative COD changes were influenced by surgical type, corneal region, and time point. At 1-week and 1-month postoperatively, COD in the 0 to 6 mm region was higher in the SMILE group compared to the FS-LASIK group, potentially due to differences in baseline COD and cap/flap thickness between the 2 procedures.

## 1. Introduction

Small incision lenticule extraction (SMILE) and femtosecond laser-assisted in situ keratomileusis (FS-LASIK) are 2 predominant refractive laser surgical techniques for correcting myopia and myopia astigmatism. Both procedures have consistently demonstrated safety, stability, and predictability in the correction of high myopia.^[1]^ These techniques correct refractive errors by reshaping the cornea. Despite differences in their surgical approaches and underlying mechanisms, they share a common goal: to enhance visual quality while minimizing the risk of surgical complications.

Corneal optical density (COD) is a critical parameter for assessing corneal transparency and overall corneal health.^[2]^ It holds numerous applications in corneal diagnostics, including the detection of bacterial keratitis, keratoconus, corneal cystine deposits, Fuchs endothelial dystrophy, among others.^[3–5]^ Additionally, COD serves as a valuable indicator for monitoring pathological changes, such as alterations in corneal stroma post-surgery, cellular activation, or haze formation following corneal procedures.^[6,7]^ Refractive corneal surgeries, involving laser-based tissue ablation and remodeling, inevitably impact the optical properties of the cornea. Previous studies have demonstrated that myopic laser refractive surgery can induce changes in COD,^[8]^ with evidence suggesting a correlation between the severity of corneal haze and the increase in COD.^[9]^ This suggests that COD may serve as a predictive marker for postoperative surgical outcomes and the quality of vision.

COD can be objectively quantified by measuring the backward scattering of light through corneal tissue using a Scheimpflug camera.^[10]^ The Pentacam Scheimpflug system provides a robust tool for evaluating corneal transparency both before and after refractive surgery.^[11,12]^ It is widely employed for the quantitative measurement of COD, representing corneal transparency across various regions using grayscale units.

While previous studies have reported changes in COD following SMILE and FS-LASIK, much of the research has primarily focused on long-term postoperative variations in COD.^[13,14]^ These studies indicate that COD tends to stabilize over time after both procedures. However, there remains a relative paucity of research on the detailed postoperative short-term changes and their influencing factors. Moreover, variations in sample size, follow-up duration, and measurement methodologies across studies have led to inconsistent findings. A comprehensive analysis of the factors influencing postoperative COD changes, particularly with respect to surgical type, preoperative corneal parameters, and surgical variables, remains insufficient. The precise manner in which these factors individually or interactively affect postoperative COD is yet to be fully elucidated. Therefore, further systematic and in-depth research is needed to explore the changes in COD and their influencing factors following SMILE and FS-LASIK.

This study aims to retrospectively analyze patients with high myopia who underwent SMILE and FS-LASIK, comparing the changes in COD across different time points and regions postoperatively. Additionally, the study seeks to explore the potential underlying causes of these changes. The findings of this study will enhance our understanding of the impact of refractive surgery on corneal tissue, provide clinicians with valuable insights for assessing surgical outcomes, and aid in the optimization of surgical strategies to offer improved visual outcomes for myopic patients.

## 2. Patients and methods

### 2.1. Patients

Data from patients who underwent SMILE and FS-LASIK procedures at the Fourth Affiliated Hospital of Soochow University between May 2021 and June 2024 were retrospectively reviewed.

The inclusion criteria for this study were: patients aged 18 years or older; best corrected visual acuity of ≥ 0.8; spherical equivalent of 6.0D or greater; and astigmatism of 3.0D or less. Additionally, all participants had no history of prior corneal refractive surgery.

Exclusion criteria included: patients diagnosed with keratoconus, suspected keratoconus, or any form of corneal ectasia; active ocular inflammation; moderate to severe dry eye disease; fundus pathology; severe adnexal eye conditions; a history of any eye surgeries; prior use of corneal contact lenses, where soft contact lenses had not been discontinued for at least 1 week, or hard contact lenses for at least 3 months; and history of ocular trauma and glaucoma.

This study was approved by the Ethics Committee of the Fourth Affiliated Hospital of Soochow University (approval No. 231020), and informed consent was obtained from all participants, in compliance with the Declaration of Helsinki.

### 2.2. Methods

The SMILE procedure was performed using the VisuMax femtosecond laser system (Carl Zeiss Meditec AG, Jena, Germany). The laser parameters included an energy setting of 135 nJ, a scanning frequency of 500 Hz, spot spacing of 4.5 μm, a cap thickness ranging from 110 to 130 μm, an optical zone diameter between 6.2 and 6.7 mm, and a base thickness of 10 to 15 μm.

FS-LASIK also utilized the VisuMax femtosecond laser system (Carl Zeiss Meditec AG, Jena, Germany) for corneal flap creation, with a flap thickness ranging from 90 to 110 μm, and the flap hinge positioned at the 12 o’clock position. Corneal stromal bed ablation was carried out using the WaveLight EX500 excimer laser (Alcon Laboratories, Fort Worth), with an optical zone diameter ranging from 6.2 to 6.7 mm. Ablations were centered on the corneal apex using the Custom Q protocol. All surgeries were performed by a single, experienced surgeon, and all patients received the same treatment approach.

To measure COD, the Pentacam Scheimpflug imaging system (Oculus Optikgerate GmbH, Wetzlar, Germany) was employed. During measurements, patients were positioned with their head against the device, and their natural pupil was focused in a dark room. The focus was manually adjusted prior to scanning, and 3 measurements were taken for each patient, with the average value recorded. Only data that met the acceptable quality criteria were included in the analysis. These measurements were performed by a single, experienced physician.

The recorded data included slit lamp examination findings, as well as preoperative (pre) and postoperative COD values at 1-day (pos1d), 1-month (pos1m), 3-months (pos3m), and 6-months (pos6m) following surgery.

#### 2.2.1. Statistical analysis

Statistical analyses were performed using SPSS Statistics 19.0 (IBM, Armonk) and Prism 7.0 for Windows (GraphPad Software, La Jolla). The normality of the data was assessed using the Kolmogorov–Smirnov (K–S) test. Metric data that followed a normal distribution were expressed as mean ± standard deviation. The mixed-effect linear model was adopted in multivariate analysis to avoid the effect of binocular data. Changes in COD across different time points and regions within the SMILE and FS-LASIK groups were analyzed using multivariate repeated measures analysis. Between-group comparisons of COD were conducted using Student *t* test. The relationship between the difference in COD between the 2 groups and various corneal characteristics was explored using Pearson correlation analysis. These correlation parameters were subsequently incorporated into a stepwise variable selection multivariate regression analysis to identify the final influencing factors. Statistical significance was set at a *P* value of <.05.

## 3. Results

### 3.1. Participants

This study included a total of 279 eyes, with 132 eyes undergoing SMILE and 147 eyes undergoing FS-LASIK. No statistically significant differences were observed in the patients’ preoperative baseline characteristics between the 2 groups (Table 1, all *P* > .05). However, significant differences were noted in cap/flap thickness, lenticule thickness or ablation depth, suction time, and residual stromal thickness (RST) between SMILE and FS-LASIK (all *P* < .001). The ablation zone showed no significant differences between the 2 groups.

**Table 1:** Patient preoperative and intraoperative baseline characteristics.

Characteristics	SMILE group (n = 132 eyes)		FS-LASIK group (n = 147 eyes)		*P*
Preoperative	Mean ± SD	Range	Mean ± SD	Range	
Age (yr)	24.79 ± 5.43	18 to 40	24.36 ± 5.54	18 to 43	.66
Gender (male/female)	58/74	–	64/83	–	.52
Eye (R/L)	65/67	–	72/75	–	.53
BCVA (logMAR)	−0.04 ± 0.04	−0.10 to −0.18	−0.04 ± 0.05	−0.10 to −0.18	.76
bIOP (mm Hg)	15.45 ± 2.19	10.7 to 22.2	15.51 ± 2.15	12.2 to 22.6	.66
Spherical error (D)	7.40 ± 1.06	−8.75 to −6.00	7.58 ± 1.21	−9.25 to −6.25	.22
Cylinder (D)	0.95 ± 0.56	−2.50 to 0.00	1.04 ± 0.72	−3.25 to 0.00	.34
SE (D)	7.87 ± 1.10	−9.00 to −6.13	8.10 ± 1.26	−9.88 to −6.25	.12
Flat K (D)	42.23 ± 1.37	39.40 to 45.10	42.59 ± 1.26	38.90 to 45.40	.07
Steep K (D)	43.87 ± 1.37	41.10 to 46.50	44.02 ± 1.39	41.10 to 46.60	.38
Q value	−0.29 ± 0.15	−0.59 to 0.18	−0.27 ± 0.12	−0.52 to 0.11	.29
Angle kappa	0.16 ± 0.09	0.01 to 0.53	0.17 ± 0.09	0.02 to 0.46	.21
WTW (mm)	11.7 ± 0.43	10.70 to 12.60	11.6 ± 0.35	10.90 to 12.50	.42
MCT (μm)	540.13 ± 18.79	517 to 608	533.38 ± 29.27	480 to 583	.13
Baseline COD					
0–2 mm region (GSU)	16.27 ± 1.00	14.04 ± 18.98	16.38 ± 0.92	13.66 ± 18.85	.32
2–6 mm region (GSU)	14.47 ± 0.87	11.98 ± 16.69	14.55 ± 0.72	12.82 ± 16.90	.40
6–10 mm region (GSU)	14.00 ± 2.19	8.49 ± 20.49	14.26 ± 2.10	9.29 ± 18.94	.30
10–12 mm region (GSU)	27.43 ± 4.56	18.34 ± 39.96	26.98 ± 4.45	18.35 ± 36.27	.37
Intraoperative					
Cap/Flap thickness (μm)	118.63 ± 7.38	110 to 130	101.96 ± 8.40	90 to 110	<.001*
Ablation zone (mm)	6.46 ± 0.16	6.2 to 6.7	6.46 ± 0.11	6.2 to 6.7	.94
LT/AD (μm)	134.83 ± 8.75	92 to 148	113.56 ± 15.80	55 to 145	<.001*
Suction time (s)	28.65 ± 0.74	27 to 30	21.88 ± 1.21	20 to 23	<.001*
RST (μm)	299.35 ± 17.14	280 to 360	308.46 ± 11.69	280 to 360	<.001*

AD = ablation depth, BCVA = best corrected visual acuity, bIOP = biomechanically corrected intraocular pressure, D = diopters, FS-LASIK = femtosecond laser-assisted in situ keratomileusis, GSU = grayscale units, K = keratometry, LogMAR = logarithm of the minimum angle of resolution, LT = lenticule thickness, MCT = minimum corneal thickness, RST = residual stromal thickness, SE = spherical equivalent, SMILE = small incision lenticule extraction, WTW = white-to-white.

* *P* < .05, significant difference between the SMILE and FS-LASIK groups.

All surgeries were successfully performed without vision threatening complications, and all patients were followed up for at least 6-months.

### 3.2. Changes in COD

**Figure 1.**
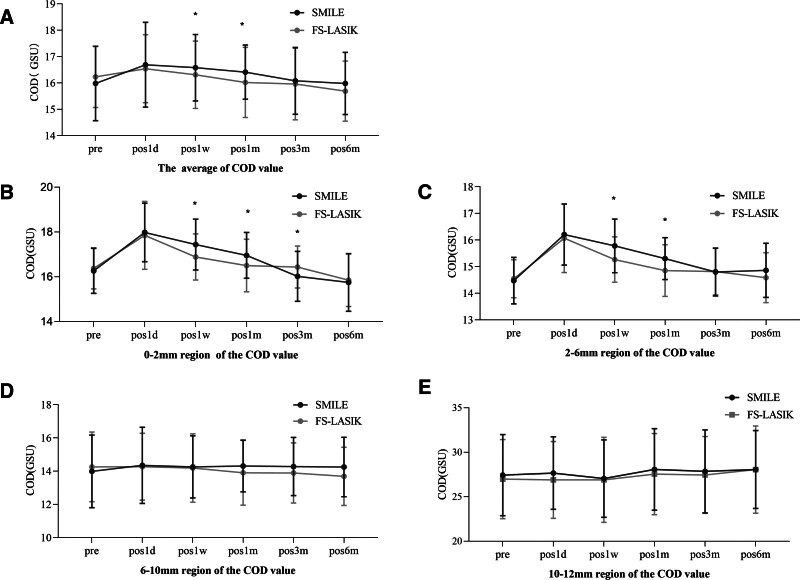
Changes in COD at 1 day, 1 week, 1 month, 3 months, and 6 months after SMILE and FS-LASIK. (A) Changes in average COD values at each postoperative time point. (B) Changes in COD values in the 0–2 mm corneal region at each postoperative time point. (C) Changes in COD values in the 2–6 mm corneal region at each postoperative time point. (D) Changes in COD values in the 6–10 mm corneal region at each postoperative time point. (E) Changes in COD values in the 10–12 mm corneal region at each postoperative time point. *P < .05, statistically significant difference. COD = corneal optical density, d = day, FS-LASIK = femtosecond laser-assisted in situ keratomileusis, GSU = gray scale units, m = month, pre = preoperative, SMILE = small incision lenticule extraction, w = week.

The postoperative changes in COD for both SMILE and FS-LASIK were illustrated in Figure 1.

### 3.3. Trend of COD changes

The average COD in both groups exhibited a downward trend at the 6-month postoperative follow-up.

### 3.4. Subgroup analysis

Based on distinct corneal regions, we further classified the data into subgroups. Repeated measures analysis of variance was conducted to assess the COD values at different regions and time points for both the SMILE and FS-LASIK groups. In the mixed-effects model, postoperative changes in COD were significantly influenced by the type of surgery (*F*_type_ = 5.649, *P* < .05), corneal region (*F*_region_ = 12.991, *P* < .001), and time point (*F*_time_ = 11.558, *P* < .001).

### 3.5. Comparison between the 2 groups

At 1-week and 1-month postoperatively, significant differences in the average COD were observed between the 2 groups. As shown in Figure 1A, the SMILE group exhibited higher COD values compared to the FS-LASIK group at these time points (1-week: 16.58 ± 1.26 vs 16.31 ± 1.28, *P* < .05; 1-month: 16.41 ± 1.03 vs 16.02 ± 1.33, *P* < .05).

### 3.6. Regional and temporal comparisons

Regional differences: COD values in the 0 to 2 and 2 to 6 mm regions were higher in the SMILE group compared to the FS-LASIK group, whereas no differences were observed in the 6 to 12 mm region (Fig. 1B–E).

Temporal differences: in the 0 to 2 mm region, the SMILE group exhibited higher COD values at 1-week and 1-month postoperatively (1-week: 17.44 ± 1.13 vs 16.89 ± 1.03, *P* < .05; 1-month: 16.96 ± 1.02 vs 16.50 ± 1.17, *P* < .05). At 3-months postoperatively, the COD values in the SMILE group was lower than in the FS-LASIK group (16.02 ± 1.12 vs 16.44 ± 0.94, *P* < .05). In the 2 to 6 mm region, the SMILE group demonstrated higher COD values at both the 1-week and 1-month postoperative time points (1-week: 15.78 ± 1.01 vs 15.27 ± 0.85, *P* < .05; 1-month: 15.30 ± 0.79 vs 14.85 ± 0.97, *P* < .05; Fig. 1B–E).

### 3.7. Factors influencing changes in COD

#### 3.7.1. SMILE group, 0 to 2 mm region

Correlation analysis revealed that, at 1-week postoperatively, COD was positively correlated with baseline COD (*r* = 0.723, *P* < .001), cap thickness (*r* = 0.319, *P* < .05), RST (*r* = 0.329, *P* < .05), and angle kappa (*r* = 0.312, *P* < .05). At 1-month, COD was also positively correlated with baseline COD (*r* = 0.671, *P* < .001), RST (*r* = 0.278, *P* < .05), and angle kappa (*r* = 0.333, *P* < .05).

Multiple linear regression analysis identified baseline COD and cap thickness as significant risk factors for COD changes at 1-week (β = 0.868, *P* < .001; β = 0.018, *P* < .05). Baseline COD was also a significant risk factor for COD changes at 1-month (β = 0.734, *P* < .001).

#### 3.7.2. SMILE group, 2 to 6 mm region

In the 2 to 6 mm region, COD at 1-week was positively correlated with baseline COD (*r* = 0.670, *P* < .001), cap thickness (*r* = 0.266, *P* < .05), RST (*r* = 0.297, *P* < .05), and angle kappa (*r* = 0.275, *P* < .05). At 1-month, COD was positively correlated with baseline COD (*r* = 0.690, *P* < .001), RST (*r* = 0.301, *P* < .05), and angle kappa (*r* = 0.304, *P* < .05).

Multiple linear regression analysis identified baseline COD as a risk factor for COD changes at both 1-week (β = 0.805, *P* < .001) and 1-month (β = 0.641, *P* < .001).

#### 3.7.3. FS-LASIK group, 0 to 2 mm region

In the 0 to 2 mm region, postoperative COD at 1-week was positively correlated with baseline COD (*r* = 0.294, *P* < .05) and flap thickness (*r* = 0.287, *P* < .05). At 1-month, COD remained positively correlated with baseline COD (*r* = 0.288, *P* < .05) and flap thickness (*r* = 0.268, *P* < .05).

Multiple linear regression analysis indicated that baseline COD and flap thickness were significant risk factors for COD changes at 1-week (β = 0.168, *P* < .05; β = 0.012, *P* < .05). Baseline COD was also identified as a significant risk factor for COD changes at 1-month (β = 0.226, *P* < .05).

#### 3.7.4. FS-LASIK group, 2 to 6 mm region

In the 2 to 6 mm region, postoperative COD at 1-week was positively correlated with flap thickness (*r* = 0.337, *P* < .001), minimum corneal thickness (*r* = 0.304, *P* < .05), and baseline COD (*r* = 0.261, *P* < .05). At 1-month, COD was positively correlated with baseline COD (*r* = 0.417, *P* < .001), flap thickness (*r* = 0.274, *P* < .05), and central corneal thickness (*r* = 0.230, *P* < .05).

Multiple linear regression analysis identified flap thickness as a significant risk factor for COD changes at 1-week (β = 0.015, *P* < .001). Baseline COD was a significant risk factor for COD changes at 1-month (β = 0.312, *P* < .001).

## 4. Discussion

In the present study, we first observed that the change in COD within the 0 to 6 mm zone at 1-week and 1-month postoperatively was higher in the SMILE group compared to the FS-LASIK group. Additionally, our research identified several factors influencing COD changes, including baseline COD and cap/flap thickness. These findings underscore the critical role of preoperative corneal evaluation in predicting postoperative outcomes and optimizing surgical planning.

Our results indicate a decreasing trend in the average COD for both the SMILE and FS-LASIK groups during the 6-month postoperative follow-up. This observation is consistent with the findings of Wei et al and Shajari et al,^[14,15]^ who reported a gradual recovery and stabilization of corneal transparency following refractive surgery. Similarly, Han et al, through long-term follow-up, observed comparable changes in COD post-surgery.^[13]^ The reduction in COD can be attributed to the corneal healing process,^[14]^ which likely involves the resolution of inflammation, remodeling of the extracellular matrix, and recovery of corneal endothelial cells.^[16]^

It is noteworthy that our study identified several significant factors influencing COD changes, including the type of surgery, corneal region, and postoperative duration. Statistically significant differences in COD were observed between the SMILE and FS-LASIK groups, with the SMILE group showing higher COD values in the 0 to 2 and 2 to 6 mm regions at 1-week and 1-month postoperatively. This observation suggests that the 2 surgical techniques, along with the resulting corneal remodeling, may affect the optical properties of the cornea differently, thereby influencing the changes in corneal transparency. Kim et al demonstrated, through observation of corneal thickness and curvature changes during a 3-month follow-up, that corneal remodeling patterns vary between SMILE and FS-LASIK procedures.^[17]^ The higher COD observed in the SMILE group, particularly in the early postoperative period, may be due to multiple factors. First, the cap thickness in SMILE is generally greater than the flap thickness in FS-LASIK. In our study, the SMILE cap thickness was concentrated around 120 µm, while the FS-LASIK flap thickness averaged 100 µm, which could contribute to increased light scattering and higher COD values. Second, the lenticule extraction process in SMILE may induce more mechanical stress on the corneal tissue, leading to corneal edema or microscopic structural changes that temporarily increase COD.^[18]^ Finally, differences in laser energy settings and scanning patterns between the 2 procedures may also contribute to the observed COD differences. Agca et al used confocal microscopy to compare changes in posterior stromal backscatter after SMILE and FS-LASIK, revealing that, although there was an initial increase in anterior stromal backscatter, the increase was more significant in the early postoperative period in the SMILE group and gradually returned to baseline levels, further supporting the notion that the surgical technique influences corneal transparency.^[19]^

Our study also revealed regional and temporal variations in COD. The central corneal region (0–2 mm) exhibited the most significant changes, followed by the para-central region (2–6 mm), while the peripheral region (6–12 mm) showed minimal changes. This observation aligns with the findings of Cai et al, who noted that changes in the central region were most pronounced in SMILE and LASEK procedures, while the peripheral region showed the least change.^[20]^ The reasons for these regional differences may stem from the denser arrangement of collagen fibers in the central cornea compared to the peripheral cornea, with anisotropy in fibril packing potentially influencing transparency and refractive index.^[21]^ Furthermore, the lenticule extracted in SMILE is a convex lens, with greater thickness in the center and thinner edges, whereas the laser ablation depth in FS-LASIK is deeper in the central region than in the peripheral region. Therefore, both surgical techniques involve more substantial ablation in the central cornea than in the peripheral region, which could explain why the central region (0–2 mm) shows the most significant changes. The lack of significant changes in the 6 to 12 mm region may indicate that peripheral corneal tissue, which is less involved in the surgery, undergoes relatively similar healing patterns in both groups. However, a study by Poyales F, et al^[22]^ reported similar changes in COD between FS-LASIK and SMILE in different regions, which differs from our findings and may be attributable to the study’s focus on subjects with low to moderate myopia.

Regarding the temporal changes, the SMILE group exhibited higher COD values in the 0 to 2 and 2 to 6 mm regions at 1-week and 1-month postoperatively, with a gradual decrease over time. This result can be explained by the slight interface opacity due to the corneal stromal healing reaction following laser surgery, which gradually recovers to normal transparency over time with the administration of anti-inflammatory medications.^[23]^ This may also provide an explanation for the prolonged visual recovery in the SMILE group compared to FS-LASIK in terms of corneal transparency.^[24]^ By the 6-month follow-up, no significant differences were observed between the 2 groups. In a study by Wei et al, it was found that at 6 to 12 months postoperatively, no significant differences in COD were observed between the SMILE and FS-LASIK groups.^[14]^ However, in the early postoperative period, FS-LASIK showed faster recovery of corneal transparency, particularly in the central region, which may be due to the thinner flap in FS-LASIK compared to SMILE. Our multivariate regression analysis also confirmed that cap thickness is an important factor influencing COD changes. Liu et al also found that a thinner cap resulted in better early postoperative visual quality.^[25]^ Alio et al, using optical coherence tomography, observed that SMILE corneas exhibited higher optical density than LASIK corneas in the early postoperative period, which then significantly decreased by 3 months. These findings are consistent with the delayed visual recovery observed in SMILE.^[26]^

Our study identified several factors influencing COD changes, including baseline COD and cap/flap thickness. These findings emphasize the importance of preoperative corneal characteristics and surgical parameters in predicting postoperative COD changes. Patients with higher baseline COD may be more susceptible to increased COD after surgery. These patients may benefit from more conservative surgical parameters or closer postoperative monitoring to minimize potential visual disturbances. Previous studies have shown a correlation between normal COD and age,^[27,28]^ with optical density increasing with age.^[29]^ The correlation between COD and age may be attributed to the reduction of endothelial cells, which are crucial for maintaining corneal clarity.^[30]^ While age was consistent with baseline COD as a factor influencing COD changes, our study population was predominantly around 24 years old, with no significant age differences between the 2 groups, and therefore, age was not analyzed as a factor in our study. Li et al explored the distribution of optical density across different corneal depths and related factors, such as corneal curvature and astigmatism, which may also influence postoperative COD changes.^[31]^ However, our study did not find a significant impact of corneal curvature or astigmatism on COD, and further studies with larger sample sizes are needed to confirm these findings. Additionally, corneal flap/cap thickness may affect COD changes by influencing the wound healing process, corneal biomechanics, and corneal curvature, which in turn affects corneal remodeling.^[17,29]^

There are several clinical implications of our findings. First, our study highlights the necessity of personalized surgical planning based on preoperative corneal characteristics to optimize postoperative outcomes. Second, our study provides a basis for counseling patients regarding the expected postoperative course and potential visual disturbances. Finally, identifying risk factors for increased COD can guide postoperative management strategies, such as the use of topical corticosteroids or artificial tears to promote faster recovery and reduce complications.

This study has some limitations. First, the sample size was relatively small, and the follow-up period was limited to 6 months. Larger-scale studies with longer follow-up periods are needed to confirm our findings and explore the long-term changes in COD. Second, we did not conduct a detailed assessment of surgical parameters (such as laser energy settings and scanning patterns) on COD changes. Future research should investigate these factors for a more in-depth understanding of their impact on postoperative corneal optical properties. Lastly, attention should be paid to the long-term stability of COD changes and their relationship with visual outcomes.

In conclusion, our study demonstrates that SMILE and FS-LASIK surgeries have distinct effects on COD in patients with high myopia. Surgical type, corneal region, and postoperative duration are significant factors influencing COD changes. Identifying risk factors for increased COD can guide preoperative counseling, surgical planning, and postoperative management strategies to optimize patient outcomes. Future studies should explore the long-term changes in COD and the impact of surgical parameters on postoperative corneal optical properties.

## Acknowledgments

We would like to thank our colleagues and collaborators for their valuable support and assistance in this study.

## Author contributions

**Conceptualization:** Jing Lou, Li Wang, Yun Wang.

**Data curation:** Jing Lou, Yue Xu, Yun Wang.

**Formal analysis:** Jing Lou.

**Funding acquisition:** Yao Xu.

**Investigation:** Jing Lou, Yao Xu.

**Methodology:** Jing Lou, Yue Xu, Yun Wang, Yao Xu.

**Project administration:** Jing Lou, Yue Xu, Yao Xu.

**Software:** Jing Lou, Li Wang, Yun Wang, Xiaofeng Zhang.

**Supervision:** Jing Lou, Li Wang, Yun Wang, Xiaofeng Zhang.

**Validation:** Li Wang, Xiaofeng Zhang.

**Visualization:** Xiaofeng Zhang.

**Writing – original draft:** Jing Lou, Xiaofeng Zhang.

**Writing – review & editing:** Jing Lou.
